# Beyond Malignancy: Clinical Insights from Three Cases of Severe Hypercalcemia

**DOI:** 10.3390/jcm15062412

**Published:** 2026-03-21

**Authors:** Shani Ben Dori, Noor Kabaha, Amer Abu Husseine, Eilam Rabina, Liat Barzilay Yoseph, Pnina Rotman-Pikielny, Martin H. Ellis, Osnat Jarchowsky Dolberg

**Affiliations:** 1 Department of Internal Medicine A, Meir Medical Center, 59 Tchernichovsky St, Kfar Saba 4428164, Israel; 2 Cardiology Department, Meir Medical Center, Kfar Saba 4428164, Israel; 3 Endocrinology Institute, Meir Medical Center, Kfar Saba 4428164, Israel; 4 Gray School of Medicine, Faculty of Medical and Health Sciences, Tel Aviv University, Tel Aviv 6997801, Israel; 5 Hematology Institute, Meir Medical Center, Kfar Saba 4428164, Israel

**Keywords:** hypercalcemia, primary hyperparathyroidism, Addison’s disease, PTH-related protein, lactation

## Abstract

Severe hypercalcemia is a life-threatening condition requiring immediate treatment alongside a systematic evaluation to identify the underlying cause. Although malignancy is a common etiology among hospitalized patients, alternative causes must be considered to guide targeted therapy, as illustrated in these cases. The first case involved a 31-year-old postpartum woman with corrected calcium levels of 14.5 mg/dL and suppressed PTH. Hypercalcemia resolved after tapering and temporary cessation of breastfeeding, consistent with lactation-associated hypercalcemia that is likely PTHrP-mediated. The second case describes a 30-year-old woman who presented with hypotension, hypercalcemia, hyperphosphatemia, and low PTH. A systematic evaluation revealed severe glucocorticoid deficiency consistent with primary adrenal insufficiency (Addison’s disease). The final case featured a 47-year-old man with severe symptomatic hypercalcemia (18.5 mg/dL) and markedly elevated PTH. Imaging revealed a 3 cm parathyroid tumor. Selective parathyroidectomy produced a rapid intraoperative PTH decline, and pathology supported parathyroid adenoma rather than carcinoma. Together, these cases highlight that symptomatic severe hypercalcemia is a medical emergency warranting prompt clinical intervention, followed by an early PTH-based stratification to direct a focused, stepwise diagnostic workup and definitive management.

## 1. Introduction

Severe hypercalcemia is a life-threatening condition that requires immediate treatment followed by a systematic evaluation to identify and manage the underlying cause. Hypercalcemia is a common metabolic disorder with diverse etiologies. Primary hyperparathyroidism is the leading cause in the general population, with a prevalence of 1–2 per 1000 adults. In patients with malignancy, hypercalcemia occurs in approximately 2–3% of cases and may reach up to 30% in certain tumor types and advanced stages. While primary hyperparathyroidism predominates in the outpatient setting, malignancy-associated hypercalcemia is the most common cause among hospitalized patients and those presenting with hypercalcemic crisis [1,2,3]. Severe hypercalcemia is a life-threatening emergency that can rapidly progress to multi-organ dysfunction. Neurologically, it causes confusion, stupor, and coma; renally, it leads to acute kidney injury and irreversible renal damage; cardiac involvement presents as life-threatening arrhythmias and arrest due to shortened QT interval; gastrointestinal effects such as nausea, vomiting, and pancreatitis worsen dehydration; and musculoskeletal complications include muscle weakness and pathological fractures. In hypercalcemic crisis, this rapid multi-organ failure carries high mortality, making immediate intervention essential for preventing fatal outcomes [4].

In this case series, we present three hospitalized patients with profound hypercalcemia from non-malignant etiologies, emphasizing the importance of considering alternative causes to ensure accurate diagnosis and guide targeted therapy.

### 1.1. Patient No. 1: Case Presentation

A 31-year-old primiparous woman with a history of endometriosis, approximately 4 weeks postpartum after delivery at 38 weeks and 5 days’ gestation and currently breastfeeding, was referred following routine blood tests that revealed hypercalcemia, with an albumin-corrected serum calcium levels of 14.5 mg/dL (normal range: 8.5–10.5 mg/dL) and concomitant renal impairment with serum creatinine of 1.5 mg/dL (baseline: 0.8 mg/dL). She had no relevant family medical history. She was asymptomatic and denied any regular medication, except for a once-daily multivitamin providing approximately 400 IU of vitamin D. Physical examination was unremarkable, with no evidence of lymphadenopathy or palpable breast masses. Electrocardiogram (ECG) showed no abnormalities suggestive of hypercalcemia. Additional laboratory evaluation showed urea of 38 mg/dL (normal range: 10–50 mg/dL) and serum phosphate of 2.5 mg/dL (normal range: 2.5–4.5 mg/dL).

Given the severity of hypercalcemia, the treatment was initiated immediately with aggressive intravenous isotonic fluid resuscitation. Due to volume overload with peripheral edema during rehydration, a single dose of furosemide was administered. Following initial stabilization, further laboratory workup revealed suppressed parathyroid hormone (PTH) levels at 7.4 pg/mL (normal range: 15–65 pg/mL), mildly elevated 25-hydroxyvitamin D [25(OH)D] at 130 nmol/L (normal range: 76–125 nmol/L), along with normal 1,25-dihydroxyvitamin D [1,25(OH)_2_D] levels (148 pmol/L; normal range 47.8–190.3 pmol/L), consistent with the ingestion of vitamin D.

### 1.2. Patient No. 2: Case Presentation

A 30-year-old woman with a known history of Hashimoto’s thyroiditis presented to our institution with progressive weakness, nausea, vomiting, and abdominal pain for the past 3 weeks. The patient had been on levothyroxine replacement therapy for hypothyroidism, with a recent dose escalation of 100 mcg once daily with TSH 9.05 mIU\L for 2 months prior to presentation. She denied fever, night sweats, weight loss, or other constitutional symptoms.

On presentation, she was hypotensive with sinus tachycardia and was afebrile. Physical examination was without other remarkable findings.

Initial laboratory studies revealed hypercalcemia (12 mg/dL) (normal range: 8.5–10.5 mg/dL), hyperphosphatemia (6.2 mg/dL) (normal range: 2.5–4.5 mg/dL), hypoglycemia (62 mg/dL) (normal range: 70–100 mg/dL), and normal renal function creatinine of 0.5 mg/dL (baseline: 0.8 mg/dL).

Intravenous isotonic fluid resuscitation was promptly instituted, alongside comprehensive laboratory investigations, which revealed PTH of <10 pg/mL (normal range: 15–65 pg/mL), 25-hydroxyvitamin D [25(OH)D] of 54.7 nmol/L (normal range: 76–125 nmol/L), and 1,25-dihydroxyvitamin D [1,25(OH)_2_D] of 5 pmol/L (normal range 47.8–190.3 pmol/L).

### 1.3. Patient No. 3: Case Presentation

A 47-year-old man with a history of heavy smoking was admitted due to fatigue, anorexia, and unintentional weight loss, reflected by a body mass index (BMI) decrease from 24.5 to 22.5 kg/m^2^. On physical examination, he appeared cachectic and generally unwell without additional remarkable findings.

The initial laboratory tests revealed profound hypercalcemia with acute kidney injury. The albumin-corrected serum calcium level was 18.5 mg/dL (normal range: 8.5–10.5 mg/dL), and serum creatinine was 1.74 mg/dL, increased from a baseline of 0.8 mg/dL. Serum phosphate was 2.3 mg/dL (normal range: 2.5–4.5 mg/dL). Immediate therapy included aggressive intravenous fluids, calcitonin (250 units twice daily for 48 h), and a single 5 mg dose of intravenous zoledronic acid. PTH was markedly elevated at 490 pg/mL (normal range: 15–65 pg/mL). 25-hydroxyvitamin D [25(OH)D] was 23.1 nmol/L (normal range: 76–125 nmol/L), and 1,25-dihydroxyvitamin D [1,25(OH)_2_D] was 14.2 pmol/L (normal range: 37.8–190.3 pmol/L), consistent with vitamin D deficiency. Accordingly, oral vitamin D supplementation was initiated.

## 2. Discussion

**Figure 1:**
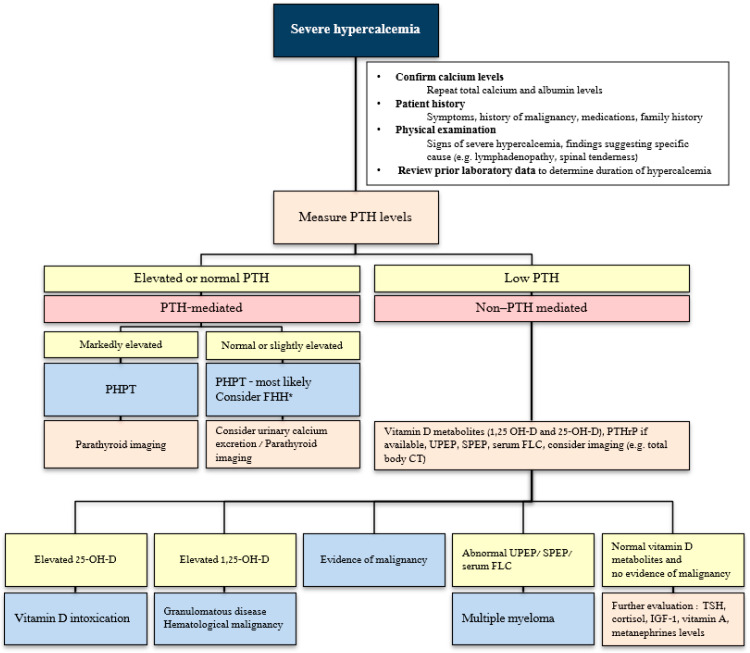
Suggested stepwise algorithm for hypercalcemia [4,5]. * Based on the patient’s history and laboratory findings, evaluation for familial hypocalciuric hypercalcemia should be performed. PTH—parathyroid hormone; PHPT—primary hyperparathyroidism; FHH—familial hypocalciuric hypercalcemia; PTHrP—parathyroid hormone-related protein; 1,25 OH-D—1,25 dihydroxyvitamin D; 25 OH-D—25-hydroxyvitamin D; UPEP—urine protein electrophoresis; SPEP—serum protein electrophoresis; FLC—free light chains; TSH—thyroid-stimulating hormone; IGF-1—insulin-like growth factor 1.

The evaluation of hypercalcemia begins with the confirmation of hypercalcemia and the measurement of PTH levels, which distinguishes between PTH-mediated and non-PTH-mediated causes. PTH-mediated causes are most often due to primary hyperparathyroidism, whereas non-PTH-mediated hypercalcemia encompasses a wide spectrum of disorders, including malignancy, vitamin D-related conditions, granulomatous diseases, endocrinopathies, medication effects, and rare genetic syndromes (Table 1). In cases of non-PTH-mediated hypercalcemia, further evaluation should be guided by the patient’s clinical presentation. As illustrated in Figure 1, the diagnostic evaluation is typically conducted in a stepwise manner. However, in the context of hypercalcemic crisis, diagnostic tests, namely the confirmation of hypercalcemia, measurement of intact PTH, and assessment for common non-PTH-mediated causes, should be performed concurrently and should not delay initiation of life-saving treatment [4,5].

### 2.1. Patient No. 1: Diagnosis

A comprehensive evaluation was undertaken to investigate non-PTH-dependent etiology of hypercalcemia. After aggressive intravenous hydration, the cessation of potential contributors (including vitamin D-containing supplements) and a recommendation to discontinue breastfeeding improved renal function. A contrast-enhanced whole-body CT scan was performed only after renal recovery and did not demonstrate lymphoproliferative disease or solid malignancy. Serum free light chains, immunofixation, and protein electrophoresis were all within normal limits, excluding multiple myeloma. Thyroid function was normal. Further endocrine evaluation included assessment for acromegaly and adrenal insufficiency. Serum insulin-like growth factor 1 (IGF-1) levels were normal, and morning cortisol was 16.7 µg/dL, indicating normal adrenal function (Table S1 in Supplementary Materials).

Non-malignancy parathyroid hormone-related protein (PTHrP)-mediated hypercalcemia during pregnancy and lactation has been described and is thought to reflect physiologic PTHrP secretion from the placenta during pregnancy and the mammary gland during lactation periods, which can rarely become clinically significant [6,7,8]. In our patient, PTHrPs were ultimately within the normal range (2.3 pmol/L, reference range <4.2 pmol/L). Unfortunately, for logistic reasons, the ordering of this test was delayed and the results obtained did not reflect the hormone levels during lactation. Following gradual reduction and temporary cessation of breastfeeding, calcium normalized and PTH became mildly elevated, compatible with prior reports in which hypercalcemia improves after weaning or after reduced milk production [6,7].

Beyond PTHrP, defects in vitamin D catabolism have emerged as an important consideration in pregnancy- and lactation-associated hypercalcemia. Loss-of-function mutations in CYP24A1 impair the degradation of active vitamin D metabolites and may precipitate or amplify hypercalcemia during pregnancy or the postpartum period, occasionally with nephrolithiasis and nephrocalcinosis [6,9]. In our case, the combination of suppressed PTH, normal 1,25-dihydroxyvitamin D, and a course tightly linked to breastfeeding (with biochemical resolution after weaning) favors lactation-associated hypercalcemia mediated by PTHrP as the most likely explanation, despite normal PTHrP levels obtained outside the lactational peak [6,7,9].

This case underscores lactation-associated hypercalcemia, a rare but increasingly recognized cause of non-PTH-mediated hypercalcemia in postpartum women. In most published lactational PTHrP cases, hypercalcemia is mild and self-limited, yet severe hypercalcemia has been documented, indicating that marked calcium elevations, as described in our patient, can occur [6,7].

### 2.2. Patient No. 2: Diagnosis

Hypercalcemia with low PTH levels prompted a systematic evaluation to identify the underlying cause of non-PTH-mediated hypercalcemia. The diagnostic workup included screening for granulomatous diseases, malignancy (total body CT, protein electrophoresis, and complete blood count), and endocrine disorders (thyroid function tests and urine metanephrines to exclude pheochromocytoma), all of which were normal.

An additional adrenal function assessment revealed significant abnormalities in adrenocortical function: morning serum cortisol was significantly decreased (0.4 ug/dL) (normal range: 138–690 nmol/L), and adrenocorticotropic hormone (ACTH): >2000 pg/mL was markedly elevated (normal range: 7.2–63.3 pg/mL) (Table S1 in Supplementary Materials).

In accordance with established clinical guidelines, a Synacthen stimulation test was deemed unnecessary, as the biochemical findings of suppressed cortisol levels in conjunction with markedly elevated ACTH were sufficient to confirm the diagnosis of primary adrenal insufficiency [10]. Upon confirmation of the diagnosis, hydrocortisone replacement therapy was promptly initiated.

The hypercalcemia observed in this case was attributed to adrenal insufficiency, as an uncommon presentation of Addison’s disease [11]. The clinical presentation may include nonspecific symptoms such as confusion, anorexia, vomiting, and dehydration, which can overlap with both adrenal crisis and hypercalcemia itself. In some cases, hypercalcemia may be the initial or predominant feature, particularly in the context of acute adrenal crisis or in patients with renal impairment [11,12].

The underlying mechanism of hypercalcemia in adrenal insufficiency is multifactorial and not yet fully elucidated. The most widely accepted contributing mechanisms include enhanced intestinal calcium absorption, impaired renal calcium excretion secondary to volume depletion and reduced glomerular filtration rate, and increased calcium mobilization from the bone. The latter has been observed but without evidence of increased bone resorption or elevated bone turnover markers, suggesting a non-PTH-mediated process [13,14]. Prompt recognition and glucocorticoid replacement are essential, as hypercalcemia typically resolves rapidly with the correction of adrenal insufficiency [14].

The American Association of Clinical Endocrinology and the American Thyroid Association note that patients with autoimmune hypothyroidism are at increased risk for primary adrenal insufficiency based on shared autoimmune pathophysiology [15]. Importantly, the initiation or dose escalation of levothyroxine in unrecognized adrenal insufficiency may precipitate or accelerate life-threatening adrenal crisis, as thyroid hormone accelerates cortisol clearance. Accordingly, both societies recommend that glucocorticoid replacement always precedes levothyroxine when adrenal insufficiency is suspected or confirmed [15].

In this case, the patient presented with accompanying hyperphosphatemia, which represents an uncommon but recognized biochemical manifestation of primary adrenal insufficiency [16]. Aldosterone deficiency impairs renal phosphate excretion while causing sodium wasting and volume depletion, reducing glomerular filtration rate and compromising phosphate clearance. Concurrent glucocorticoid deficiency further diminishes glomerular filtration and alters proximal tubular phosphate handling, culminating in elevated serum phosphate concentrations [16].

Coexistent hypothyroidism may exacerbate this effect through a reduced metabolic rate and impaired renal phosphate excretion. In addition, the initiation or dose escalation of levothyroxine may cause hyperphosphatemia in untreated adrenal insufficiency [17].

The coexistence of Addison’s disease and Hashimoto’s thyroiditis raised the clinical suspicion of autoimmune polyglandular syndrome type 2 (APS-2), prompting further autoantibody screening; 21-hydroxylase autoantibodies and thyroid peroxidase antibodies were obtained to confirm the autoimmune etiology of both conditions. Notably, no unique confirmatory test exists for APS-2 itself; the diagnosis remains entirely clinical, based on the confirmed coexistence of these autoimmune endocrinopathies in the same individual, thereby fulfilling the established diagnostic criteria for APS-2 [18].

As seen in this case, hypercalcemia and hyperphosphatemia may be the first presentation of Addison’s disease triggered by thyroid replacement therapy.

### 2.3. Patient No. 3: Diagnosis

A working diagnosis of primary hyperparathyroidism was made and neck ultrasound was performed. A hypoechoic solid lesion with a cystic component measuring 2.6 × 1.5 cm located inferior to the right thyroid lobe was identified without cervical lymphadenopathy.

**Figure 2:**
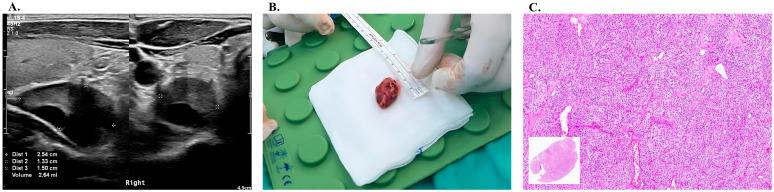
Patient’s parathyroid lesion: ultrasound, intraoperative, and histopathologic findings. (**A**) Neck ultrasonography demonstrating a hypoechoic parathyroid lesion; (+) outlines the lesion margins; (**B**) Intraoperative photograph from the patient’s parathyroidectomy; (**C**) patient’s histopathology showing a parathyroid adenoma on hematoxylin and eosin (H&E) staining.

The patient subsequently underwent surgical exploration of the neck and an enlarged parathyroid gland was identified and excised from beneath the lower pole of the right thyroid lobe. The intraoperative PTH levels dropped from 689 pg/mL pre-excision to 61 pg/mL post-excision, consistent with a complete removal of the hyperfunctioning gland (Table S1 in Supplementary Materials and Figure 2).

The primary diagnostic consideration was whether this represented an unusual presentation of a common condition—parathyroid adenoma, usually associated with mild and asymptomatic hypercalcemia—or a rare but serious cause such as parathyroid carcinoma, more often linked to marked hypercalcemia. While large cohorts demonstrate that serum calcium exceeding 12 mg/dL is distinctly uncommon in adenomas [19,20], emerging evidence suggests large adenomas account for the majority of cases presenting with severe hypercalcemia, thereby challenging the traditional paradigm that such profound elevations are most indicative of carcinoma [21].

Histopathological examination demonstrated hypercellular parathyroid tissue without evidence of local invasion to contiguous structures, and the concurrently excised lymph node was benign, which was compatible with parathyroid adenoma.

Primary hyperparathyroidism classically affects the skeleton and kidneys, and its clinical burden ranges from asymptomatic biochemical disease to end-organ complications. Skeletal manifestations include reduced bone mineral density with osteopenia or osteoporosis, increased fragility fracture risk, and, in advanced disease, osteitis fibrosa cystica with brown tumors. Renal involvement includes nephrolithiasis and nephrocalcinosis and may be accompanied by a decline in glomerular filtration rate and chronic kidney disease. Additional reported manifestations include neuromuscular and neurocognitive symptoms, gastrointestinal complaints, and cardiovascular sequelae such as hypertension and arrhythmias [22].

## 3. Conclusions

Management begins with prompt stabilization of hypercalcemic crisis, followed by a systematic, clinically guided evaluation to define the underlying mechanism, leading to targeted therapy and favorable patient outcomes. In the first case, the postpartum presentation underscored lactation- and pregnancy-related etiologies, including transient non-malignancy PTHrP-mediated and impaired vitamin D catabolism such as CYP24A1 variants. The second case highlighted primary adrenal insufficiency as a reversible endocrine cause of severe hypercalcemia. The third case illustrated that, although primary hyperparathyroidism most often occurs due to benign adenoma and typically presents with mild hypercalcemia, it can rarely manifest as profound, life-threatening hypercalcemia.

## Figures and Tables

**Table 1:** **Common causes of hypercalcemia [4,5].** PTHrP—parathyroid hormone-related protein.

**Parathyroid hormone mediated**
Primary hyperparathyroidism
Adenoma
Hyperplasia
Carcinoma
Familial hypocalciuric hypercalcemia
Tertiary hyperparathyroidism
**Non-parathyroid hormone mediated**
Hypercalcemia of malignancy
Humoral hypercalcemia
Secretion of PTHrP (e.g., Lung)
Increased calcitriol (e.g., Lymphoma)
Local osteolytic hypercalcemia
Multiple myeloma
Solid tumor metastases (e.g., lung, breast)
Vitamin D intoxication
Chronic granulomatous disorders
Sarcoidosis
Tuberculosis
Endocrinopathies
Hyperthyroidism
Acromegaly
Pheochromocytoma
Addison’s disease
Medications
Lithium
Thiazide diuretics
Excessive vitamin A
Milk-alkali syndrome
Immobilization

## Data Availability

The data presented in this study are available on request from the corresponding author. The data are not publicly available due to privacy and ethical restrictions.

## Supplementary Materials

The following supporting information can be downloaded at: https://www.mdpi.com/article/10.3390/jcm15062412/s1, Table S1: Patients’ laboratory results.
